# Bronchiolitis Obliterans Syndrome After Allogeneic HSCT: Clinical Characteristics and Survival Outcomes in a Single Center Cohort

**DOI:** 10.3390/jcm15197410

**Published:** 2026-09-24

**Authors:** Emine Merve Savaş, Nurdan Kokturk, Ahmet Alparslan Ceran, Şeyma Yıldız, Münevver İrem Kök Yılmaz, Başak Bostankolu Değirmenci, Zeynep Arzu Yeğin, Gonca Erbaş, Özlem Güzel Tunçcan, Aysun Sarıer, Zübeyde Nur Özkurt

**Affiliations:** 1 Department of Hematology, Gazi University Faculty of Medicine; Ankara 06500, Turkeyzubeydenurozkurt@yahoo.com (Z.N.Ö.); 2 Department of Pulmonology, Gazi University Faculty of Medicine; Ankara 06500, Turkey; kokturk.nurdan@gmail.com; 3 Department of Radiology, Gazi University Faculty of Medicine; Ankara 06500, Turkey; 4 Department of Infectious Diseases, Gazi University Faculty of Medicine; Ankara 06500, Turkey

**Keywords:** bronchiolitis obliterans syndrome, pulmonary chronic graft-versus-host disease, allogeneic hematopoietic stem cell transplantation, treatment response, overall survival

## Abstract

**Background/Objectives**: Chronic graft-versus-host disease (cGVHD) remains a significant complication after allogeneic stem cell transplantation. Bronchiolitis obliterans syndrome (BOS), the best characterized form of pulmonary cGVHD, represents its most severe and therapeutically challenging manifestation and is associated with poor long-term outcomes. However, data on treatment responses and survival remain limited. **Methods**: We performed a retrospective, single-center study to characterize the clinical features, treatment responses, and survival outcomes of patients with BOS following allo-HSCT. **Results**: Between January 2003 and December 2023, 28 (4.1%) of 678 transplanted patients met the National Institutes of Health (NIH) diagnostic criteria for BOS with lung scores of 2–3. The median age was 26 years (21–51), and 67.9% were male. The median time from transplantation to BOS diagnosis was 20.9 months (11.2–45.9). Median forced expiratory volume in one second (FEV1) declined from 97.5% at transplantation to 47.5% at BOS diagnosis, while median diffusing capacity for carbon monoxide (DLCO) decreased from 72% to 35% of predicted.The best observed response was partial response (PR). The overall first-line steroid-based response rate was 14.3%. In second- and subsequent-line settings, PR rates were 25% with ibrutinib, 25% with extracorporeal photopheresis, 33.3% with ruxolitinib, and 20% with mesenchymal stem cell therapy, while no responses were observed with imatinib. Median overall survival from transplantation was 90.9 months (48.4–133.5), whereas median survival from BOS diagnosis was 27.2 months (17.4–156.9). Survival after BOS diagnosis was significantly longer in patients with a prior history of acute GVHD (79.7 vs. 25.7 months; *p* = 0.026) and in those with concomitant extrapulmonary cGVHD compared with isolated BOS (79.7 vs. 7.6 months; *p* = 0.001). **Conclusions**: Treatment responses in stage 2–3 BOS were limited, and survival remained very poor, highlighting the importance of earlier recognition and more effective therapies before fibrotic progression.

## 1. Introduction

Chronic graft-versus-host disease (cGVHD) remains one of the most serious late complications of allogeneic hematopoietic stem cell transplantation (allo-HSCT), arising through complex alloimmune and autoimmune mechanisms that ultimately lead to progressive organ dysfunction, impaired quality of life, and constitute the leading cause of non-relapse mortality [1,2,3,4]. Although the incidence of cGVHD has decreased in the posttransplantation cyclophosphamide (PTCy) era, the cumulative number of cGVHD cases continues to rise and is expected to further increase in the coming years [5]. Among the various manifestations of cGVHD, pulmonary involvement is considered the most severe and treatment-refractory form, leading to irreversible functional impairment and poor long-term outcomes [6]. Bronchiolitis obliterans syndrome (BOS) is the best-characterized phenotype of pulmonary GVHD, defined as an immune-mediated fibroproliferative process that causes progressive and irreversible obstruction of the small airways [7]. BOS typically occurs at a median of 20 months post-transplant, with a prevalence of 2.5–5% in the general allo-HSCT population and up to 14% among patients with cGVHD [7]. Despite standardized diagnostic and response criteria established by the National Institutes of Health (NIH) consensus project [8], early recognition remains challenging and therapeutic strategies are limited. Conventional approaches, particularly systemic corticosteroids, frequently fail to provide durable responses, and prognosis remains poor [9,10]. There is a significant gap in the existing evidence regarding organ-specific cGVHD, particularly pulmonary GVHD. The aim of this study was to evaluate the prevalence, clinical characteristics, treatment responses, and survival outcomes of patients with BOS following allo-HSCT at our center.

## 2. Materials and Methods

### 2.1. Study Design and Patient Population

This retrospective, single-center study included patients who underwent allo-HSCT in our stem cell transplantation unit between January 2003 and December 2023. Medical records of 678 transplanted patients were reviewed, among whom 28 fulfilled the diagnostic criteria for BOS and had NIH lung GVHD scores of 2 or 3. Patients with a score of 1 were excluded because of the small number of cases and insufficient response-assessment data.

The study was primarily designed as a single-arm observational analysis of patients with established BOS, focusing on their clinical characteristics, treatment responses, and outcomes. For descriptive context, baseline demographic and transplant characteristics of patients without BOS were also summarized in Supplementary Table S1. Given the marked imbalance between the BOS and non-BOS groups, no matched comparison cohort was constructed, and between-group comparisons were considered exploratory and interpreted with caution.

### 2.2. Diagnosis and Multidisciplinary Evaluation

During post-transplant follow-up, pulmonary symptoms were assessed at each visit. Pulmonary function tests were planned at 6-month intervals until year 5; however, adherence was inconsistent, particularly during the COVID-19 pandemic, and not all scheduled assessments were completed. Patients with suspected pulmonary GVHD were systematically evaluated by our multidisciplinary GVHD council consisting of hematology, pulmonology, radiology, and infectious diseases specialists. BOS diagnosis and treatment response were assessed according to the NIH consensus criteria applicable at the time of evaluation, using the 2005 NIH criteria for patients assessed before 2014 and the 2014 NIH criteria thereafter [8,11]. Radiologic findings, pulmonary function test parameters, and clinical symptoms were evaluated together to establish the diagnosis of BOS. In patients with isolated pulmonary involvement, active infection and other clinically relevant causes of airflow obstruction were excluded through multidisciplinary assessment incorporating clinical findings, pulmonary function testing, thoracic imaging, and microbiologic evaluation. The diagnosis was therefore based on a persistent and progressive functional and clinical pattern rather than on a single episode of respiratory deterioration. Histopathologic confirmation by lung biopsy was available in one patient. Lung biopsy was not routinely performed because of its invasiveness, procedure-related risks, and limited diagnostic yield in fibrotic small-airway disease. BOS diagnosis was otherwise based on the applicable NIH consensus criteria and integrated clinical, functional, and radiologic assessment.

Response assessment was performed by the same multidisciplinary GVHD team, integrating pulmonary function test results, NIH Lung Symptom Scores, and overall clinical findings. According to the 2014 NIH response criteria, complete response was defined as normalization of FEV1 to >80% predicted, partial response as an absolute increase of ≥10 percentage points in percent-predicted FEV1 from baseline when baseline FEV1 was <70%, and progression as an absolute decrease of ≥10 percentage points in percent-predicted FEV1. When pulmonary function tests (PFTs) were unavailable, changes in the NIH Lung Symptom Score were considered. Final response classification was based on multidisciplinary clinical judgment, taking both functional and symptomatic changes into account.

### 2.3. Pulmonary Function Test

Spirometry was performed using the MasterScreen (CareFusion, Höchberg, Germany) instrument. All tests were performed consecutively on the same day, with the patient resting; the patient had rested for at least 1 h and had not smoked. The FVC maneuver was performed by an experienced technician with the patient seated upright and the nose clipped. The best of the 3 measurements in which patients took a deep inspiration followed by a forced expiration was considered. The largest observed FVC and largest FEV1 of all acceptable values were reported. In the report, prebronchodilator FEV1 (L and %), FVC (L and %), FEV1 /FVC (%), PEF (%), and FEF 25–75 (L/s) values were recorded. The single-breath-hold technique was used to measure DLCO. Hemoglobin values (g/dL) were recorded prior to measurements. The DLCO single-breath maneuver begins with several cycles of tidal breathing followed by a full exhalation to near RV. The patient then rapidly inhales the test gas mixture to full lung capacity and holds their breath for approximately ten seconds. Following the breath-hold, the patient exhales completely and rapidly; the first portion of exhaled gas is expelled, and an alveolar sample is then collected. DLCO is then calculated from the concentrations of carbon monoxide and tracer gas in the sample.

### 2.4. Endpoints

The primary endpoint of the study was treatment response. Secondary endpoints included determining the prevalence of BOS within the transplant cohort, characterizing transplant- and disease-related clinical features of affected patients, and evaluating overall survival and survival patterns according to selected clinical characteristics.

### 2.5. Statistical Analysis

Statistical analyses were performed using SPSS version 24.0. Categorical variables were summarized as frequencies and percentages, whereas continuous variables were presented as median values with interquartile ranges (IQRs). OS was defined as the time from allo-HSCT to death from any cause, while post-BOS survival was defined as the time from BOS diagnosis to death from any cause. For analyses of post-BOS prognosis, BOS diagnosis was used as the time origin. Prior acute graft-versus-host disease (aGVHD) was defined as aGVHD occurring after allo-HSCT and before BOS diagnosis, whereas concomitant extrapulmonary cGVHD was defined according to additional organ involvement present at the time of BOS diagnosis. Survival probabilities were estimated using the Kaplan–Meier method (with the 95% CI for median post-BOS survival estimated using the Brookmeyer–Crowley method based on log-log transformed confidence limits), and comparisons of prognostic factors were performed using log-rank and pairwise tests. A *p*-value of <0.05 was considered statistically significant.

## 3. Results

In the overall allo-HSCT cohort (*n* = 678), 28 patients fulfilled the diagnostic criteria for BOS with NIH lung scores of 2–3, corresponding to a prevalence of 4.1% for grade 2–3 BOS in our cohort.

Baseline and transplant characteristics of the BOS cohort are summarized in Table 1. The most common underlying diagnosis was acute myeloid leukemia (AML), accounting for 50% of cases. The median age was 26 years (21–51), and 67.9% of patients were male. Most patients received matched related donor grafts (75%), and 60.7% underwent myeloablative conditioning. Basic demographic and transplant characteristics of the non-BOS cohort were additionally reviewed for clinical context. No statistically significant differences were observed between the BOS and non-BOS groups in age or major transplant-related characteristics (Supplementary Table S1).

### 3.1. Clinical Characteristics of BOS Cohort

Clinical characteristics of patients with pulmonary GVHD are presented in Table 2. A total of 67.8% had a prior history of aGVHD, and 75% exhibited at least one additional manifestation of cGVHD, most commonly sclerotic skin involvement (28.6%) or hepatic GVHD (14.3%). Pulmonary infections before BOS diagnosis were frequent; 64.2% of patients experienced at least one episode of bacterial pneumonia, and 25% had a history of fungal pneumonia before BOS. A total of 32.1% of patients had a history of smoking. The median interval from transplantation to BOS onset was 20.9 months (11.2–45.9). After BOS diagnosis, the median follow-up was 23.3 months (8.3–73.4), and the overall post-transplant follow-up duration was 69.1 months (28.5–108.7).

Pulmonary function parameters before transplantation and at the time of BOS diagnosis are summarized in Table 3 and Figure 1. At baseline, the median FEV1 was 3.19 L (2.31–3.52) and 97.5% (83.0–104.0) of predicted, and the median FVC was 3.33 L (2.58–3.99) and 91.0% (79.0–106.3) of predicted. The median DLCO corresponded to 72% (64–80%). At the time of BOS diagnosis, the median FEV1 was 1.83 L (0.79–2.70) and 47.5% (31.0–68.8) of predicted, and the median FVC was 2.35 L (1.32–3.73) and 58.5% (44.0–82.0) of predicted. The median DLCO declined to 35% predicted (28–42%).

### 3.2. Treatment Patterns

Overall, 39.3% of patients received a single line of BOS-directed therapy, whereas 60.7% required multiple treatment lines. Among these, 21.4% were treated with two lines, 17.9% with three lines, and 21.4% with four or more lines. All patients received montelukast, azithromycin, and inhaled corticosteroids as part of our routine adjunctive treatment for BOS.

### 3.3. Treatment Responses

All 28 patients received systemic corticosteroids as first-line therapy. Of these, 26 received corticosteroids alone, while 2 received concomitant corticosteroids and ibrutinib. Among patients treated with corticosteroids alone, 3 of 26 (11.5%) achieved a partial response (PR). In the combination group, 1 of 2 patients (50%) achieved PR. Overall, the first-line steroid-based response rate was 14.3%.

Responses to second- and subsequent-line treatments are summarized in Figure 2. Ibrutinib was administered to 12 patients in the second- or later-line setting. Four patients discontinued treatment within the first three months because of toxicity. Overall, 3 of 12 treated patients (25%) achieved a partial response. Among the 8 patients who remained on treatment long enough for formal response assessment, 3 achieved a partial response (37.5%). Of the three responders, one received concomitant ruxolitinib and another received concomitant extracorporeal photopheresis. Extracorporeal photopheresis (ECP) was administered to 8 patients in the second- or later-line setting, with partial response observed in 2 patients (25%), including one who received concomitant ibrutinib. Ruxolitinib was used in 6 patients at various treatment stages, resulting in partial response in 2 patients (33.3%), one of whom was treated concomitantly with ibrutinib. Mesenchymal stem cell therapy was administered to 5 patients beyond second-line treatment, with partial response observed in 1 patient (20%). Imatinib was used in 4 patients, and no treatment responses were documented.

### 3.4. Survival Outcomes

Overall survival analyses are presented in Figure 3. When calculated from the time of transplantation, the median OS was 90.9 months (95% CI, 48.4–133.5). When calculated from the time of BOS diagnosis, the median OS was 27.2 months (95% CI, 17.4–156.9). Estimated post-BOS survival rates at 1, 2, and 5 years were 72.4% (95% CI, 50.6–85.8), 68.1% (95% CI, 46.2–82.6), and 43.3% (95% CI, 22.4–62.5), respectively.

At the last follow-up, 16 of 28 patients had died. Thirteen deaths were associated with progressive pulmonary GVHD/BOS and recurrent respiratory complications, including pneumonia and respiratory deterioration. Among these patients, one with advanced, treatment-refractory BOS had been accepted for lung transplantation but died during the prolonged evaluation and waiting process before the procedure could be performed. The remaining three deaths were attributed to underlying disease relapse (*n* = 1), hepatic GVHD complicated by liver failure (*n* = 1), and cardiac aspergillosis with decompensated heart failure (*n* = 1).

Survival outcomes were compared according to the presence of a prior history of aGVHD and concomitant extrapulmonary cGVHD at the time of BOS diagnosis. Patients with a history of aGVHD had significantly longer OS after BOS diagnosis compared with those without aGVHD (median OS, 79.7 (29.3–129.9) vs. 25.7 (1–65.8) months; log-rank *p* = 0.026) (Figure 4). Similarly, OS from the time of BOS diagnosis was significantly longer in patients with concomitant extrapulmonary cGVHD compared with those with isolated BOS (median OS, 79.7 (1–167) vs. 7.6 (1–15.6) months; log-rank *p* = 0.001) (Figure 4). Post-BOS survival did not differ significantly according to donor type. Median post-BOS survival was 26.9 months (95% CI, 11.0–156.9) in the MRD group and 56.1 months (95% CI, 3.4–79.7) in patients with other donor types (log-rank *p* = 0.472).

## 4. Discussion

Chronic graft-versus-host disease represents one of the leading causes of late non-relapse mortality following allo-HSCT [4,12,13]. Although therapeutic strategies for cGVHD have evolved over time, it remains a therapeutically challenging condition, with persistent uncertainty regarding the achievement of effective and durable clinical responses. Bronchiolitis obliterans syndrome is the pulmonary manifestation of cGVHD as defined by NIH consensus criteria. In this single-center, retrospective analysis, we provide a comprehensive characterization of patients with moderate-to-severe BOS, with particular emphasis on pulmonary function decline, treatment responses, and survival outcomes.

The reported prevalence of BOS after allo-HSCT varies widely across studies, with estimates of approximately 2–3% among all transplant recipients, while higher rates of up to 10–20% have also been reported [14,15,16]. In a cohort of 1,145 allo-HSCT recipients transplanted between 2002 and 2006, Au et al. reported an overall BOS prevalence of 5.5% [17]. In line with large registry-based analyses and studies published between 2011 and 2016, BOS prevalence as a manifestation of pulmonary cGVHD has been reported to range from approximately 4.2% to 10% among allo-HSCT recipients, with higher rates observed in cohorts with predominant peripheral blood stem cell use [18]. In our more contemporary cohort spanning the period between 2003 and 2023, the prevalence of grade 2–3 BOS was 4.1%, similar to previously reported ranges. The observed variability in BOS prevalence across studies may be related to differences in transplant eras and evolving transplant care strategies, including changes in conditioning regimens, GVHD prophylaxis approaches, stem cell source, as well as heterogeneity in diagnostic timing and interpretation of BOS criteria. Although the introduction of PTCy-based GVHD prophylaxis has been associated with a reduction in overall GVHD incidence, the true prevalence of pulmonary GVHD, including BOS, in the PTCy era remains incompletely defined.

The timing of BOS onset in our cohort was also similar to previously reported data. In our cohort, the median time from transplantation to BOS onset was 20.9 months, consistent with BOS being a late complication of allo-HSCT, as prior studies have reported median times to diagnosis ranging from approximately 9 to 18 months, including 439 days in a large single-center cohort and 273 to 547 days in registry-based analyses [7,17,18].

A substantial proportion of patients with BOS had received myeloablative TBI as part of the conditioning regimen. However, myeloablative TBI exposure did not differ significantly between the BOS and non-BOS groups in our descriptive comparison. Given the marked imbalance in group sizes and the retrospective design, our data do not allow conclusions regarding TBI as a risk factor for BOS. Previous studies have reported conflicting findings. In a contemporary single-center study of 271 allo-HSCT recipients, myeloablative TBI was associated with a higher incidence of BOS and remained an independent risk factor in multivariable analysis [19]. In contrast, some pediatric studies comparing TBI- and busulfan-based myeloablative conditioning have reported similar rates of overall pulmonary toxicity, highlighting the heterogeneity of pulmonary outcomes across transplant populations and study designs [20]. Given that TBI-based regimens are still widely used, particularly in ALL, further studies are needed to better delineate the contribution of TBI to pulmonary cGVHD in the modern transplant era.

cGVHD demonstrates an overall response rate of approximately 40% to first-line systemic corticosteroids; however, responses are consistently lower in pulmonary involvement [21]. In our cohort, the first-line steroid response rate was only 14.3%, which may be explained by irreversible progressive small airway fibrosis at NIH lung score 2–3. In pulmonary cGVHD, the inflammatory process tends to evolve early into fibrotic remodeling and structural small airway damage, resulting in a predominantly fibroproliferative and less steroid-responsive phenotype [22].

Second-line therapies demonstrated modest activity overall. In this study, response rates were 25% with Ibrutinib, 25% with ECP, 33.3% with Ruxolitinib, and 20% with MSC, while no responses were observed with Imatinib. Although response rates were generally low, they are not markedly different from those reported in the literature for established pulmonary cGVHD [23,24,25]. Notably, data on mesenchymal stem cell therapy in pulmonary cGVHD remain limited, and the partial responses observed in our cohort suggest that this approach may represent a potential therapeutic option in selected patients. Collectively, these findings underscore the critical importance of early recognition and treatment of pulmonary GVHD before irreversible fibrotic remodeling develops, as therapeutic interventions initiated at later stages appear to confer limited clinical benefit. Novel agents such as Belumosudil [26] and Axatilimab [27] have shown encouraging response rates in pulmonary GVHD. Ongoing first-line studies are currently evaluating these novel agents, reflecting the recognition that conventional approaches yield limited responses in established pulmonary disease. Given the low efficacy of later-line therapies, there is a strong rationale for moving more active and targeted agents earlier in the treatment algorithm, before irreversible fibrotic remodeling becomes dominant. Longer-term follow-up data are required to determine whether these responses translate into sustained disease control in pulmonary involvement.

In our cohort, pulmonary GVHD presenting as the first manifestation of cGVHD appeared to be associated with inferior overall survival compared with pulmonary involvement developing in the context of established multi-organ cGVHD. Consistently, patients with a prior history of aGVHD also demonstrated improved survival after BOS diagnosis compared with those without aGVHD. Although these observations should be interpreted cautiously given the limited sample size, this clinical distinction has not been well characterized in previous studies. One possible explanation may relate to delayed recognition of isolated pulmonary involvement, as respiratory symptoms are often non-specific and may not immediately prompt comprehensive evaluation in the absence of other GVHD manifestations. Consequently, diagnosis may occur at a more advanced stage of bronchiolar injury. In contrast, patients with established systemic GVHD manifestations, including those with prior aGVHD or concomitant multi-organ cGVHD, are generally under closer surveillance and frequently receiving systemic immunosuppressive therapy, which may facilitate earlier detection and intervention when pulmonary involvement emerges. Alternatively, isolated pulmonary presentation may reflect a distinct immunobiological pattern; however, this hypothesis requires confirmation in larger, prospective cohorts.

For patients with advanced, treatment-refractory pulmonary cGVHD/BOS, lung transplantation may represent an important salvage option in carefully selected candidates. In a pan-European cohort of 105 allo-SCT recipients, 1-, 3-, and 5-year graft survival rates after lung transplantation were 85%, 72%, and 67%, respectively [28]. Despite these favorable outcomes, lung transplantation is still rarely performed in patients with end-stage pulmonary GVHD, even when the underlying hematologic disease is in remission. In our cohort, several patients were referred for lung transplantation; however, only one was accepted for transplantation and died while awaiting the procedure after a prolonged evaluation process. Greater awareness and closer multidisciplinary collaboration are needed to support timely referral and improve access to transplantation for carefully selected patients with no effective treatment alternatives.

In this cohort of patients with moderate-to-severe BOS, treatment responses remained limited despite currently available therapeutic options, with partial responses achieved in at most one-third of treated patients. These findings underscore the importance of prevention, early recognition, and timely intervention before irreversible pulmonary injury develops. Given the limited reversibility of established BOS, routine pulmonary function monitoring after allo-HCT, particularly in patients with cGVHD, is essential for detecting early airflow decline before advanced functional impairment occurs. Once BOS is suspected, prompt initiation of supportive treatment with inhaled corticosteroids, azithromycin, and montelukast, together with timely escalation to systemic therapy in progressive disease, may help stabilize pulmonary function. Although evidence for BOS prevention remains limited, optimization of cGVHD prophylaxis and close surveillance of high-risk patients are likely to facilitate earlier intervention and improve long-term pulmonary outcomes [29].

This study has several limitations. Although the overall prevalence of BOS in our cohort was comparable to that reported in the literature, the study was conducted at a single center, resulting in a relatively limited sample size. In addition, patients with NIH grade 1 BOS were excluded due to the small number of cases and the lack of clearly defined response assessment in this subgroup. Finally, although baseline characteristics of the non-BOS cohort were included for descriptive context, the between-group comparisons were exploratory and should be interpreted with caution given the marked imbalance in group sizes and the limited number of BOS cases. Nevertheless, real-world data on pulmonary GVHD are still limited, and our results from this study with relatively long-term follow-up may help address an important gap in the literature and generate new hypotheses for future studies.

In conclusion, pulmonary GVHD remains a severe and difficult-to-treat complication after allogeneic hematopoietic stem cell transplantation and is associated with poor survival. Pulmonary involvement may lead to earlier irreversible organ damage compared with other manifestations of cGVHD, highlighting the importance of timely recognition. Therefore, improved strategies for earlier diagnosis, including the development of refined diagnostic criteria, together with more effective therapeutic approaches, are critically required to improve outcomes in pulmonary GVHD.

## Figures and Tables

**Figure 1:**
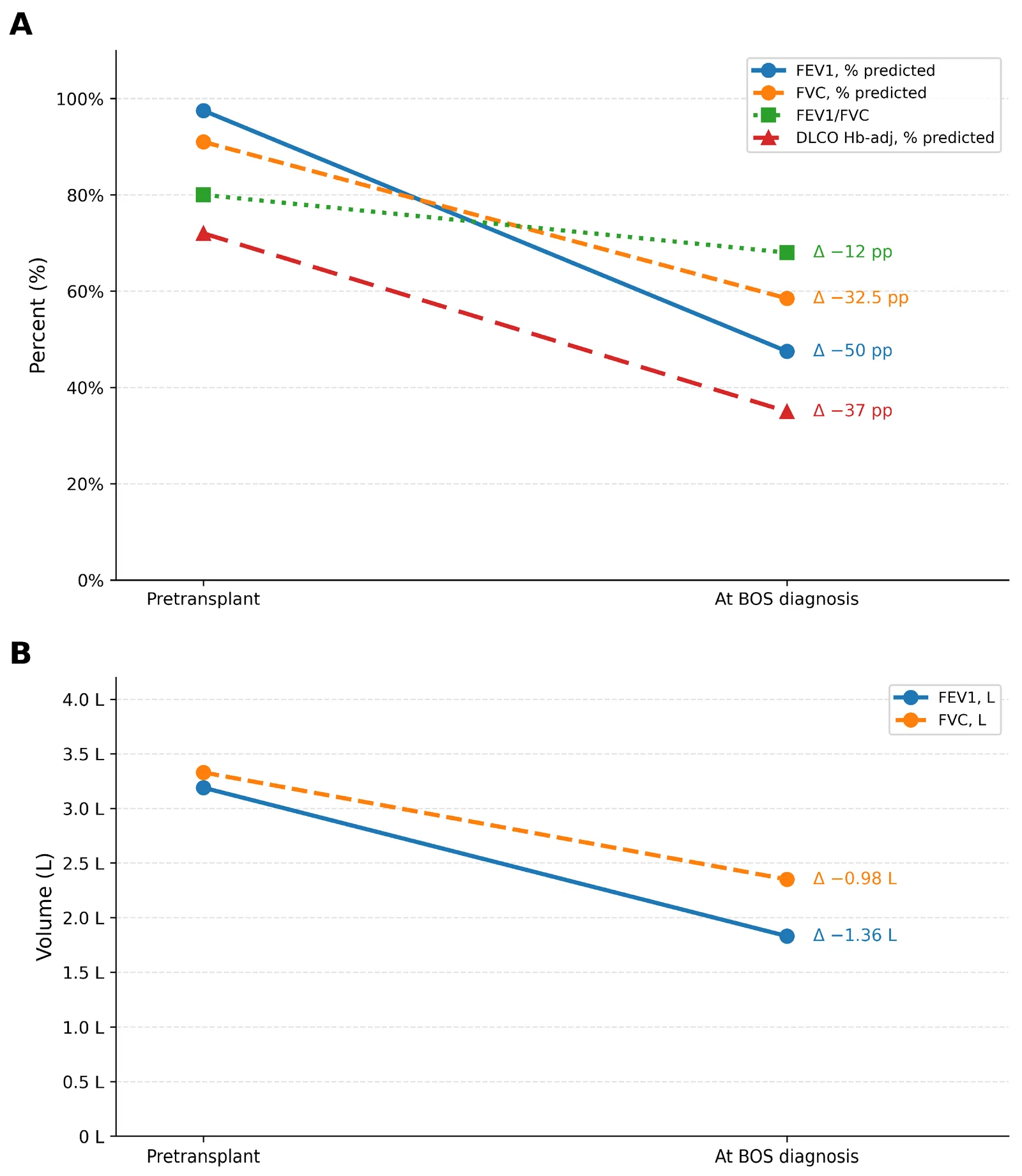
Pulmonary function parameters before transplantation and at the time of bronchiolitis obliterans syndrome (BOS) diagnosis. (**A**) Median values of forced expiratory volume in one second (FEV1), forced vital capacity (FVC), FEV1/FVC ratio, and hemoglobin-adjusted diffusing capacity of the lungs for carbon monoxide (DLCO Hb-adj), expressed as a percentage of predicted normal values. (**B**) Absolute values of FEV1 and FVC in litres. Delta (Δ) values indicate the change from pretransplant baseline to BOS diagnosis. pp, percentage points.

**Figure 2:**
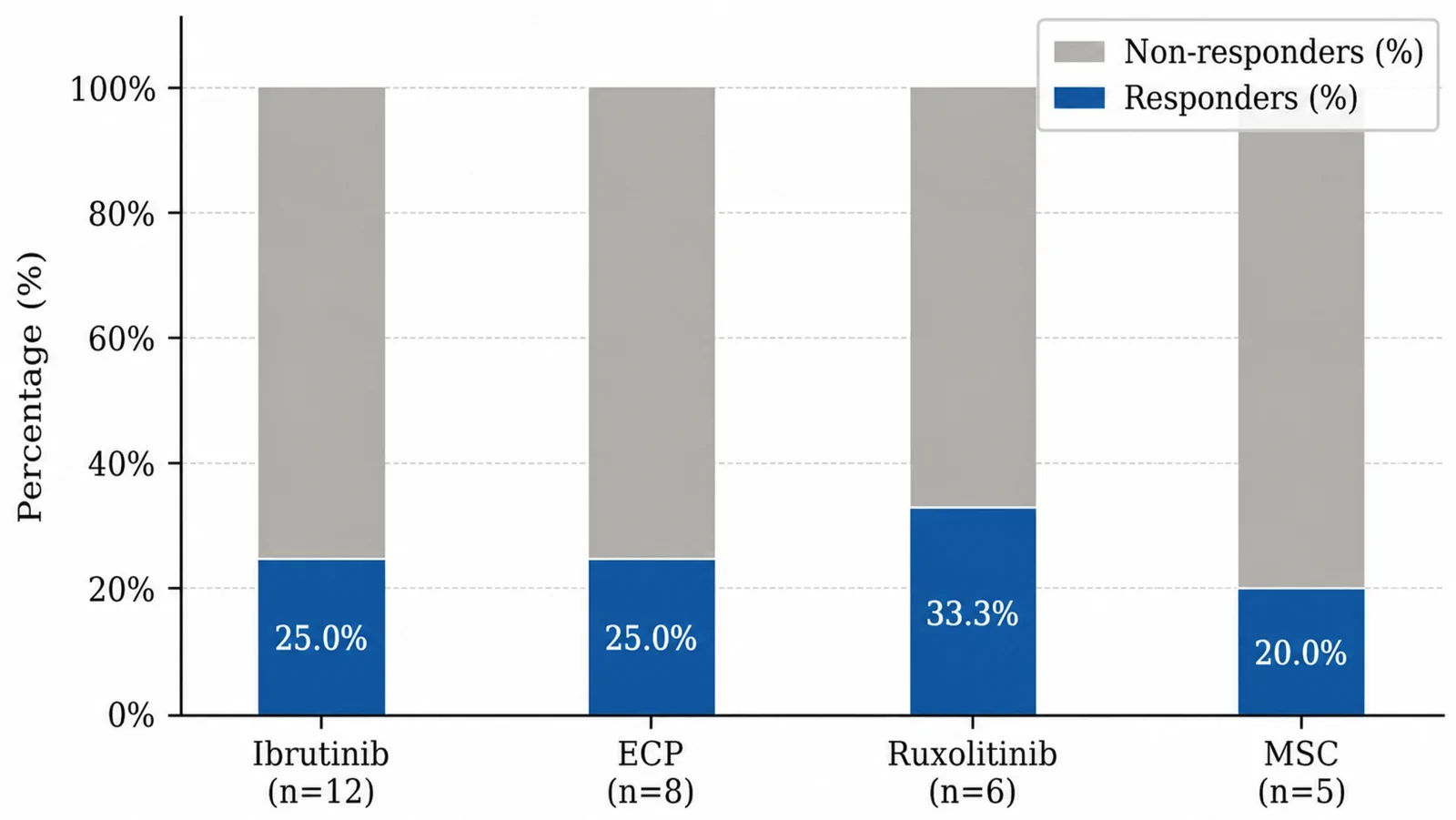
Response rates to second- and subsequent-line treatments in patients with bronchiolitis obliterans syndrome (BOS) stage 2–3. The number of patients treated with each therapy is shown in parentheses. ECP, extracorporeal photopheresis; MSC, mesenchymal stem cell therapy.

**Figure 3:**
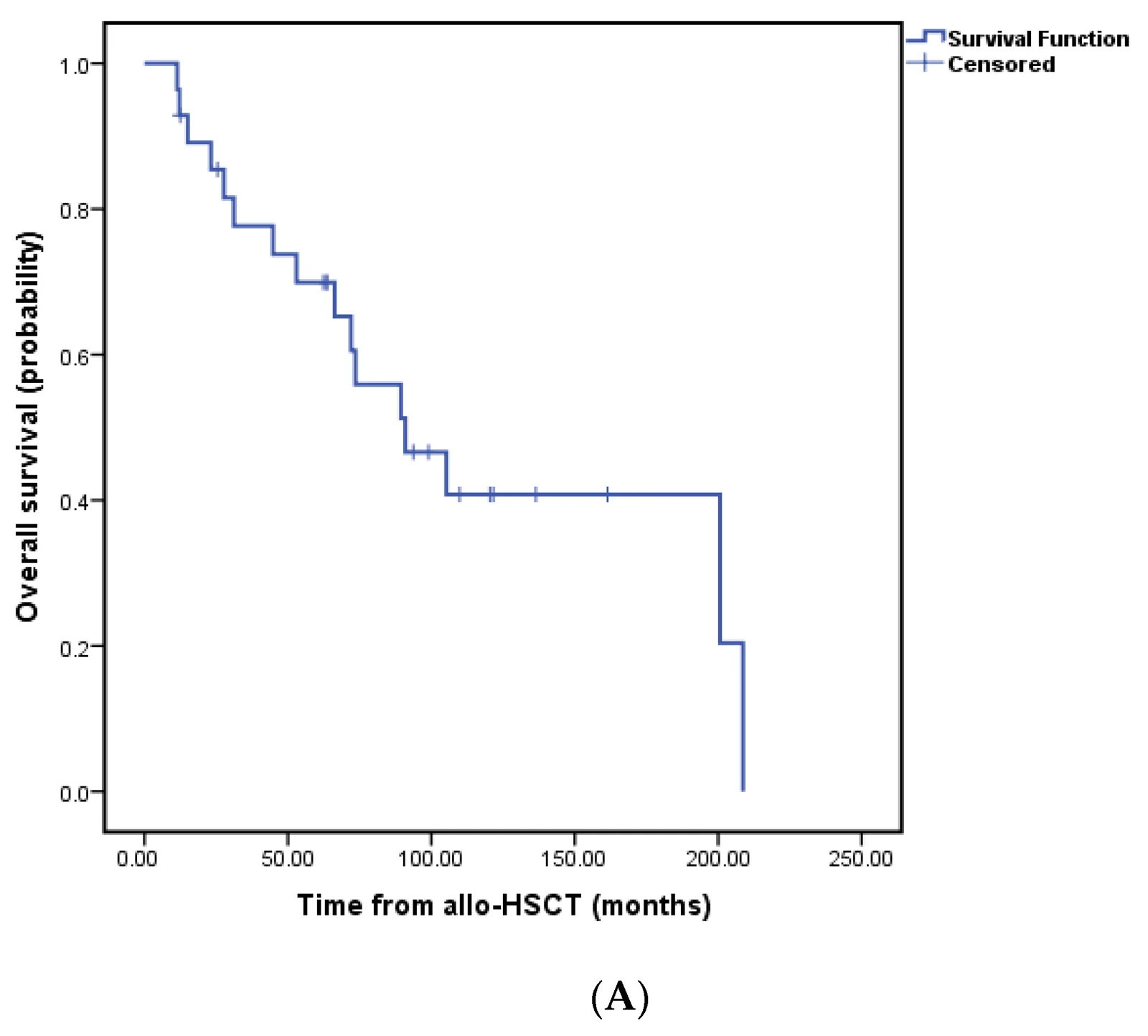

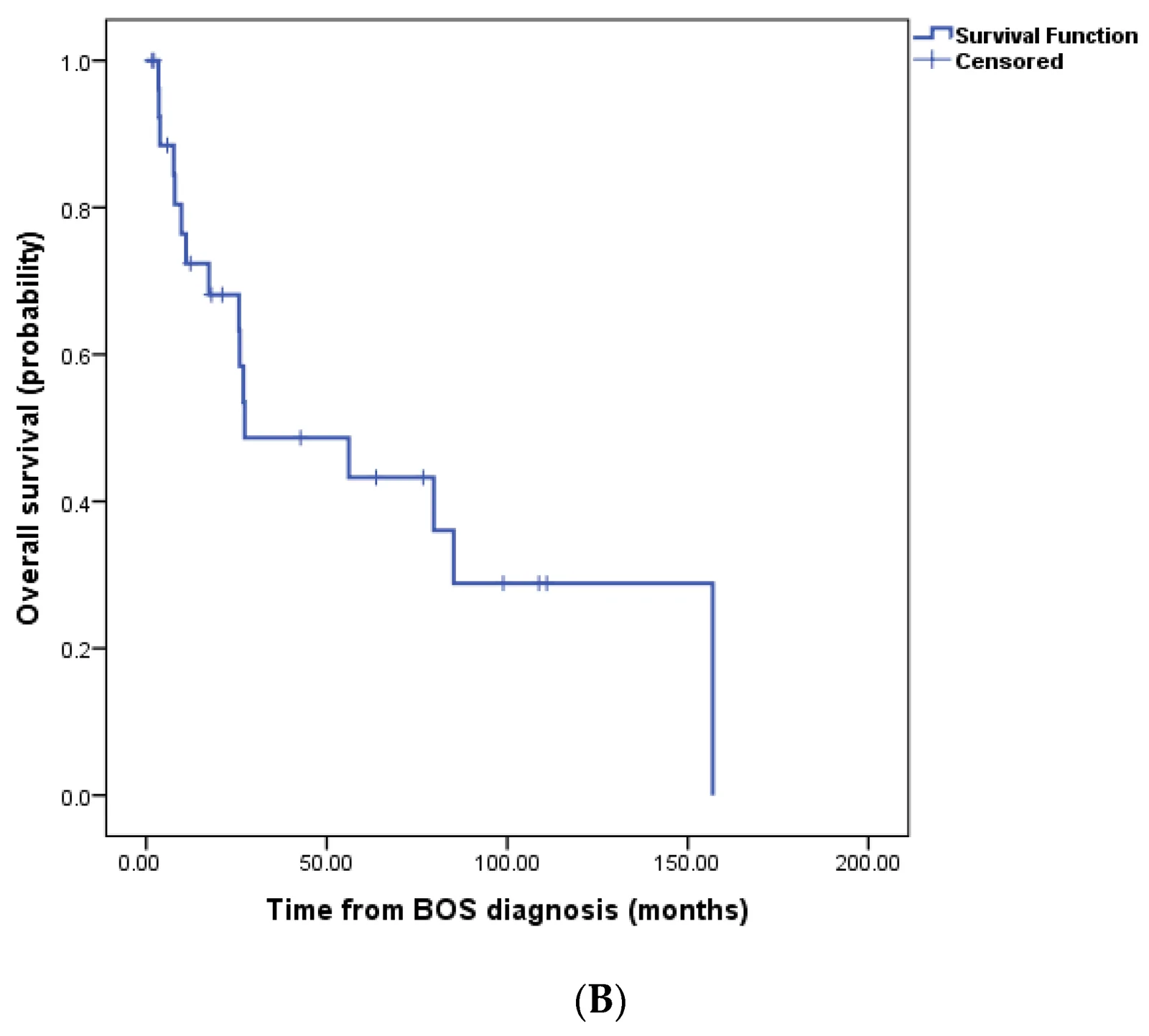
Kaplan–Meier survival curves using different time origins. (**A**) OS from allo-HSCT; (**B**) OS from BOS diagnosis.

**Figure 4:**
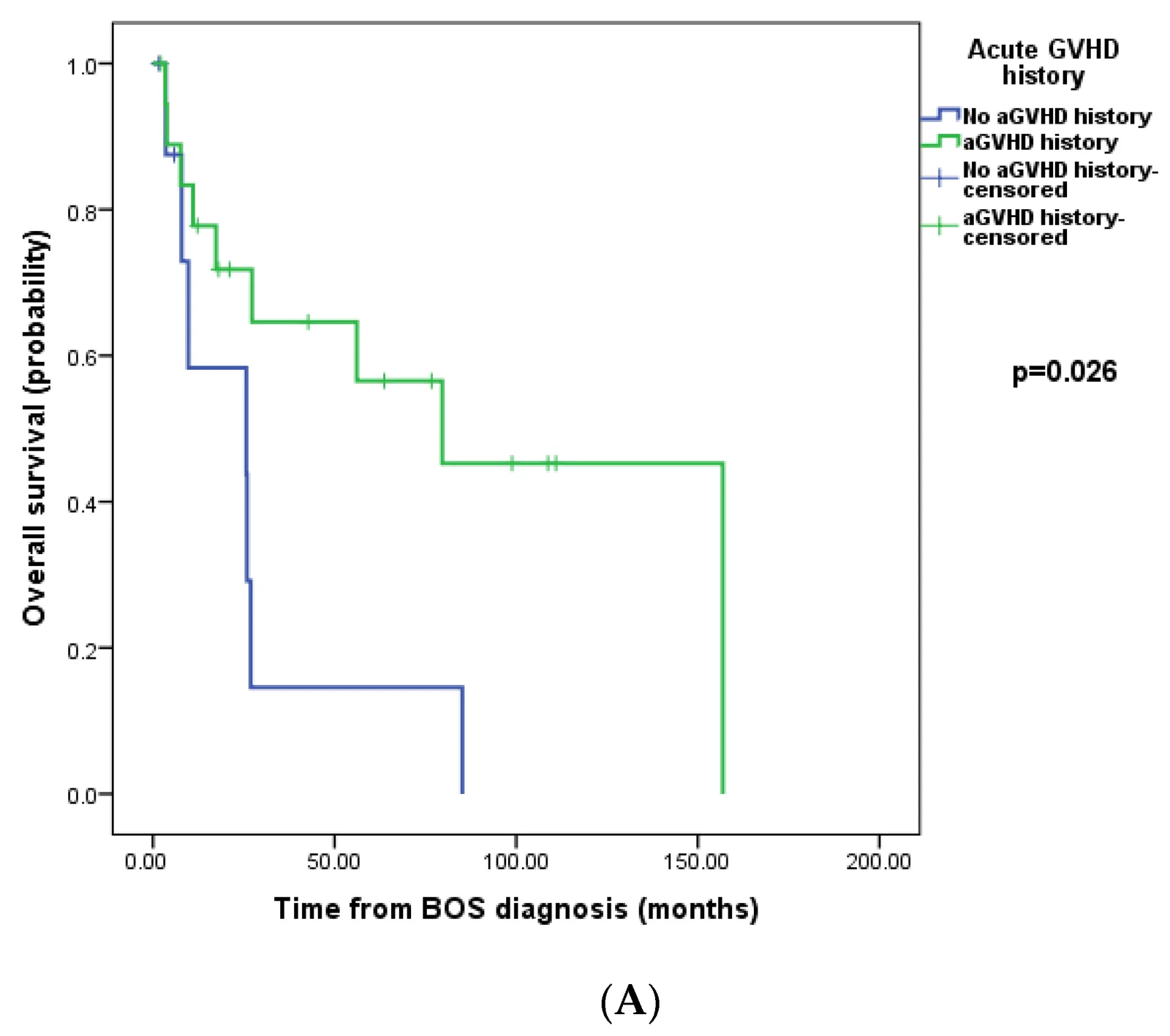

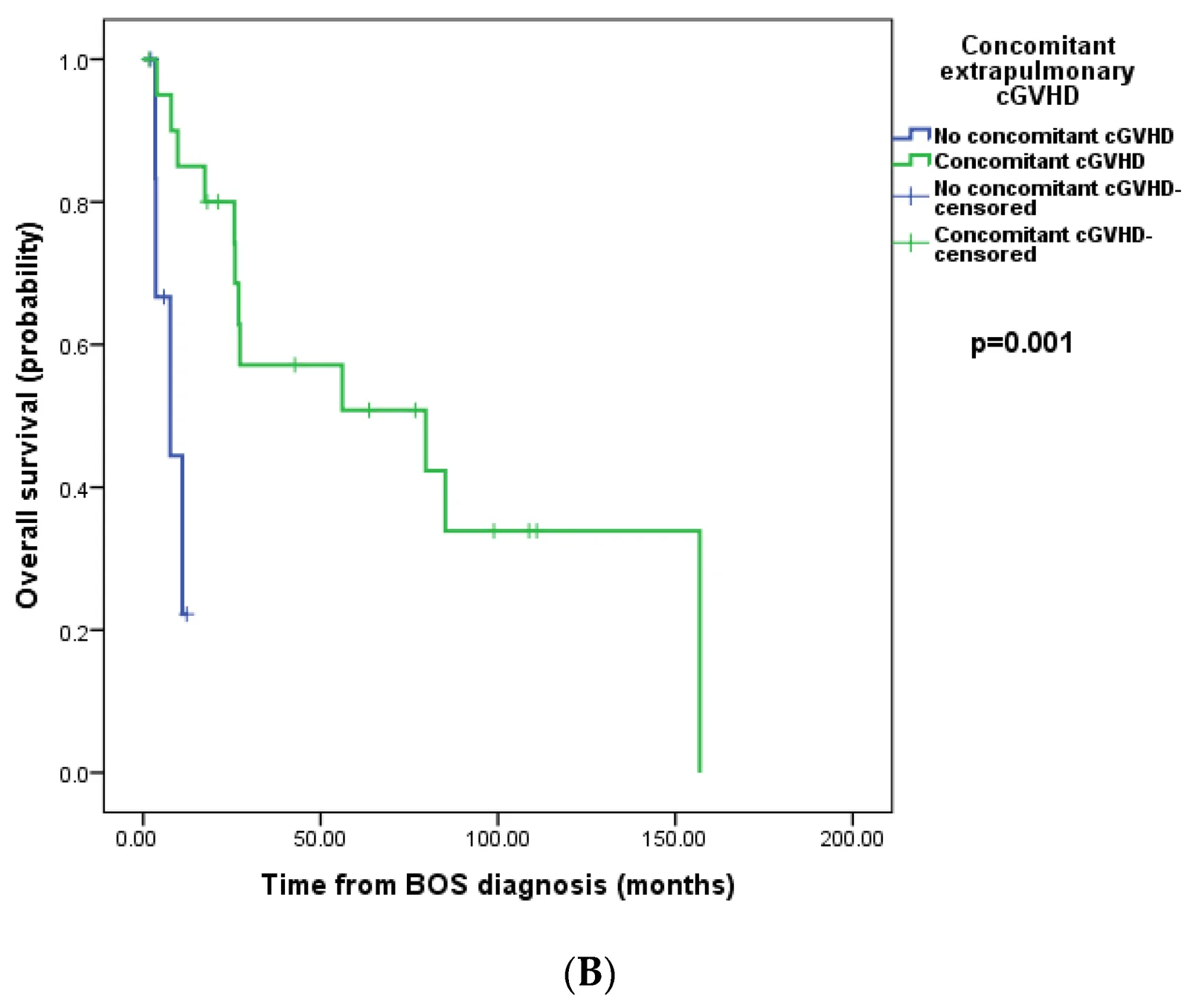
Kaplan–Meier survival curves according to clinical characteristics at BOS diagnosis. (**A**) OS according to prior aGVHD history; (**B**) OS according to concomitant extrapulmonary cGVHD.

**Table 1:** Baseline and transplant characteristics of BOS patients (*n* = 28).

Characteristic	*n* = 28
Age (years), median (IQR)	26 (21–51)
Sex, male/female, *n* (%)	19 (67.9)/9(32.1)
Diagnosis, *n* (%)	
AML	14 (50.0)
ALL	8 (28.6)
NHL	3 (10.7)
MDS	2 (7.1)
Thalassemia major	1 (3.6)
Donor type, *n* (%)	
MRD	21 (75.0)
MUD	2 (7.1)
MMUD	4 (14.3)
Haploidentical	1 (3.6)
Conditioning regimen, *n* (%)	
Myeloablative	17 (60.7)
RIC/Non-myeloablative	11 (39.3)
Myeloablative TBI (12 Gy) exposure, *n* (%)	6 (21.4)
Busulfan-based conditioning, *n* (%)	11 (39.3)
CD34^+^ cell dose (10^6^/kg), median (IQR)	4.0 (3.7–5.0)
GVHD prophylaxis (primary), *n* (%)	
CSA + MTX	17 (60.7)
CSA + MMF	11 (39.3)
GVHD prophylaxis (secondary), *n* (%)	
ATG	2 (7.1)
PTCy	1 (3.6)
Relapse, *n* (%)	2 (7.1)
Overall survival, months (95% CI)	90.9 (48.4–133.5)

Abbreviations: ALL, acute lymphoblastic leukemia; AML, acute myeloid leukemia; ATG, antithymocyte globulin; CSA, cyclosporine A; GVHD, graft-versus-host disease; MDS, myelodysplastic syndrome; MMF, mycophenolate mofetil; MMUD, mismatched unrelated donor; MRD, matched related donor; MTX, methotrexate; MUD, matched unrelated donor; NHL, non-Hodgkin lymphoma; PTCy, posttransplantation cyclophosphamide; RIC, reduced-intensity conditioning; TBI, total body irradiation.

**Table 2:** Clinical Characteristics of patients with pulmonary GVHD (BOS).

	*n* = 28
Prior acute GVHD, *n* (%)	19 (67.8)
Additional chronic GVHD manifestation, *n* (%)	21 (75.0)
Skin	8 (28.6)
Liver	4 (14.3)
Skin + ocular + liver	3 (10.7)
Skin + oral	2 (7.1)
Skin + ocular	1 (3.6)
Oral + ocular	1 (3.6)
Liver + ocular + oral	1 (3.6)
Renal	1 (3.6)
Infectious history before BOS diagnosis, *n* (%)	
Bacterial pneumonia	18 (64.2)
Fungal pneumonia	7 (25.0)
Smoking history, *n* (%) *	9 (32.1)
Time from transplant to BOS onset, months, median (IQR)	20.9 (11.2–45.9)
Post-BOS follow-up, months, median (IQR)	23.3 (8.3–73.4)
Total post-transplant follow-up, months, median (IQR)	69.1 (28.5–108.7)

* Smoking history was assessed based on smoking status before allo-HSCT.

**Table 3:** Pulmonary function parameters before transplantation and at the time of BOS diagnosis.

Parameter Median (IQR)	Pretransplant	At BOS Diagnosis
FEV1, L	3.19 (2.31–3.52)	1.83 (0.79–2.70)
FEV1, % predicted	97.5 (83.0–104.0)	47.5 (31.0–68.8)
FVC, L	3.33 (2.58–3.99)	2.35 (1.32–3.73)
FVC, % predicted	91.0 (79.0–106.3)	58.5 (44.0–82.0)
FEV1/FVC	80(75.3–91.4)	68 (62.5–87.2)
DLCO Hb-adj, % predicted	72 (64–80)	35 (28–42)

Abbreviations: BOS, bronchiolitis obliterans syndrome; DLCO, diffusing capacity of the lungs for carbon monoxide; FEV1, forced expiratory volume in one second; FVC, forced vital capacity; Hb-adj, hemoglobin-adjusted.

## Data Availability

The data that support the findings of this study are available from the corresponding author upon reasonable request.

## Supplementary Materials

The following supporting information can be downloaded at: https://www.mdpi.com/article/10.3390/jcm15197410/s1. Table S1: Baseline demographic and transplant characteristics of patients with and without bronchiolitis obliterans syndrome (BOS).
