# Supplementary material for: Bronchiolitis Obliterans Syndrome After Allogeneic HSCT: Clinical Characteristics and Survival Outcomes in a Single Center Cohort
Source: J Clin Med. 2026 Sep 24;15(19):7410. doi: 10.3390/jcm15197410 (PMC13665736; doi:10.3390/jcm15197410)
Supplement: Supplementary file 1 [file jcm-15-07410-s001.zip › jcm-4515959-supplementary.pdf]

Table S1: Baseline demographic and transplant characteristics of patients with and without bronchiolitis obliterans syndrome (BOS)

	BOS (n=28)	Non-BOS (n=650)	p
**Age, years, median (IQR)**	26 (21–51)	40 (26–52), n=649	0.055
**Male sex, n (%)**	19 (67.9)	408 (62.8)	0.691
**Diagnosis, n (%)**			0.059
AML	14 (50.0)	254 (39.1)	
ALL	8 (28.6)	143 (22.0)	
NHL	3 (10.7)	46 (7.1)	
MDS	2 (7.1)	40 (6.2)	
Thalassemia major	1 (3.6)	6 (0.9)	
Other	0	161 (24.8)	
**Donor type, n (%)**		n=637	0.467
MRD	21 (75.0)	415 (65.1)	
MMSD	0	36 (5.7)	
MUD	2 (7.1)	44 (6.9)	
MMUD	4 (14.3)	72 (11.3)	
Haploidentical	1 (3.6)	70 (11.0)	
**Busulfan-based conditioning, n (%)**	11 (39.3)	212 (32.6)	0.538
**Myeloablative TBI n (%)**	6 (21.4)	106 (16.3)	0.440
**$CD34^+$ cell dose ($\times 10^6$/kg), median (IQR)**	4.0 (3.7–5.0),	4.41 (4.0–5.0)	0.331
**Primary GVHD prophylaxis, n (%)**		n=643	0.756
CSA + MTX	17 (60.7)	424 (65.9)	
CSA + MMF	11 (39.3)	215 (33.4)	
**PTCy-containing prophylaxis, n (%)**	1 (3.6)	85 (13.1)	0.240
**ATG-containing prophylaxis, n (%)**	2 (7.1)	30 (4.6)	0.386
